# Application of Herbal Medicines with Heat-Clearing Property to Anti-Microinflammation in the Treatment of Diabetic Kidney Disease

**DOI:** 10.1155/2019/6174350

**Published:** 2019-06-02

**Authors:** Jing Guo, Yabin Gao, Yaoxian Wang, Wei Jing Liu, Jingwei Zhou, Zhen Wang

**Affiliations:** ^1^Renal Research Institution, Key Laboratory of Chinese Internal Medicine of Ministry of Education and Beijing, Dongzhimen Hospital Affiliated to Beijing University of Chinese Medicine, Beijing 100700, China; ^2^Department of Nephrology, Dongzhimen Hospital Affiliated to Beijing University of Chinese Medicine, 5 Haiyuncang, Dongcheng District, Beijing 100700, China

## Abstract

Diabetic kidney disease (DKD) is a global pandemic, and microinflammation has been reported as an important pathogenic factor of DKD. Traditional Chinese Medicine (TCM) has been used in the treatment of DKD for thousands of years, and modern Chinese medicine studies have found that herbal medicines with heat-clearing property have a curative anti-inflammation effect in DKD. This article reviews the new clinical and experimental progress made in herbal medicines with heat-clearing property, in the treatment of DKD, as well as their safety aspects.

## 1. Introduction

According to 8th edition of the International Diabetes Federation (IDF) Diabetes Atlas 2017, 8.8% of adults aged from 20 to 79 years live with diabetes mellitus (DM), estimated to be 425 million people worldwide. By 2045, the number is projected to increase to 629 million, representing 9.9% of the world population. China has the largest number of people with DM (114.4 million) among all the IDF regions [1]. DKD is one of the common complications of DM, and its onset rises with the prevalence of DM. Consolidated data from 54 countries shows that more than 80% of end-stage renal disease (ESRD) is caused by DM and high blood pressure, or both. The percentage of ESRD due to DM alone ranges from 12% to 55%, and the prevalence of ESRD in DM patients is 10 times higher than in nondiabetics [2]. In China, the line graph of the prevalence of DKD looks very steep, especially DKD with type 2 diabetes mellitus (T2DM), whose prevalence among community patients is 30%-50%, from 2009 to 2012 [3]. As a global societal catastrophe, DKD is a major financial burden to both the government and families. Therefore, it is urgent to seek effective therapeutic methods to delay or even reverse the onset and progression of DKD.

As a unique and integrated theoretical system, TCM has good clinical effects in the treatment of DKD. Evidence demonstrates that TCM can ease clinical symptoms related to DKD and improve laboratory indices such as proteinuria and serum creatinine [4–6]. In addition to tonic herbs, reports from evidence-based medicine and expert consensus show that heat-clearing herbs are also effective and essential in treating DKD [7–9], proving the theoretical hypothesis of TCM that heat evil plays a vital role in the pathogenesis of DKD. Clinical symptoms of heat syndrome in TCM, such as sore throat, halitosis, and sweating, correspond to microinflammation in the field of Western medicine [10], and according to modern pharmacological research, herbs with heat-clearing property are confirmed to have anti-inflammation and detoxification effects [11]. Recently, the relation between the progress of DKD and microinflammation has been further recognized and studied. This article will review the literature with regard to the application of heat-clearing herbs in DKD from the perspective of inflammation, making the potential advantages of TCM well understood by international researchers.

## 2. The Pathogenesis of DKD from Two Different Perspectives

### 2.1. Heat Evil as an Important Pathogen in DKD from the TCM Perspective

Heat evil is an important pathogen in TCM. It can be divided to external-heat and inner-heat evil. External heat is mostly caused by the natural evil, such as wind, cold, and dampness, which progresses into heat. For example, upper respiratory infection is similar to external heat with wind. The heat evil mentioned here refers to inner-heat evil, mostly caused by* Qi* stagnation, hyperactivity of* Yang*, and accumulation of phlegm, food, and blood stasis. The heat evil for internal disease usually includes dry heat, damp heat, stagnated heat, accumulated heat, blood heat, and static heat. Based on previous clinical experience,* Qi *and* Yin* deficiency was considered to be the core pathogenesis of DM and its complications. However, following the change in dietary and lifestyle habits, metabolic disorders and microinflammation are increasingly common in obese and diabetic patients, so researchers renewed their understanding of the pathogenesis of DM and DKD, by reviewing ancient literature. According to the* Emperor's Canon of Internal Medicine (ECIM), Xiaoke* (the TCM diagnosis for DM) can be induced by heat evil in the stomach and intestine, which leads to a series of pathogenic complications. This heat evil mainly focuses on heat stagnation and accumulation, which is associated with symptoms such as obesity, fatigue, hot flashes, halitosis, constipation or sticky stools, yellow tongue coating, and slippery pulse. After investigating 2518 obese patients with T2DM, 74.3% of them were found to have heat evil accumulation in the middle Jiao, which was considered to be a core aspect in the pathogenesis of T2DM [12]. Zhao [13] emphasized that heat evil is present throughout the progression of DM, and in line with the theory mentioned in* ECIM *that excess heat depleted* Qi *in the body, he stated that* Qi* and* Yin* deficiency in DM was secondary to the heat evil which was prevalent throughout. Prof. Zhou [14] proposed that heat evil due to blood stasis was the main pathogenic factor in DM and DKD.

Heat evil is initially manifested as dry heat, which presents as symptoms such as dry mouth, thirst, and dry skin or as damp heat, which presents as obesity, fatigue, hot flashes, halitosis, constipation, or sticky stools. Over time, the accumulation of heat evil leads to blood stasis, along with* Qi* obstruction and* Yin* deficiency. Heat evil and blood stasis then damage meridians and collaterals, resulting in clinical complications, such as numbness in the limbs, blurred vision, sharp pain, dark or purple tongue, and rough pulse. Specifically, heat evil also invades renal collaterals and combines with dampness, phlegm, and blood stasis to form micro-conglomerations in the renal collaterals, which is akin to the growth of Kimmelstiel-Wilson nodule in the pathology of DKD, according to Western medicine.

Our team [15] found that over 70% of patients with DKD had internal heat syndrome, regardless of the disease stage. In addition, our research showed that glomerular filtration rate (eGFR) had a positive correlation with heat syndrome scores in the early stage of DKD and a negative correlation in the late stage, which suggested that heat evil could be the cause for hyperfiltration and hyperperfusion in early stages of DKD, as well as the decline of renal function in late stages of DKD. Similar to our result, Wang Gang [16] reported that damp heat syndrome was considered to be the top syndrome based on a TCM syndrome analysis of 500 patients with DKD. Another research [17] showed that the frequency of prescriptions with heat-clearing herbs in the early stage of DKD was second to that of deficiency-nourishing herbs and herbs that improve blood circulation. These suggested that heat evil was a core pathogenic factor in DKD, and the administration of TCM herbs with heat-clearing property was imperative in the treatment of DKD.

### 2.2. Microinflammation as an Important Pathogenesis Process in DKD from the Perspective of Western Medicine Theory

Microinflammation involves the accumulation of inflammatory cells and overexpression of adhesion molecules, chemokines, and proinflammatory cytokines, along with the participation of pathways such as the janus kinase/signal transducers and activators of transcription (JAK/STAT) pathway, which is common in obesity, DM, and DKD. This process is different from inflammation due to infection and is termed “microinflammation” to distinguish both types [18].

Hyperglycemia is the main factor in the progression of DM to DKD. In addition, the metabolic alteration associated with accumulation of toxic products like advanced glycation end products (AGEs) and the activation of renin-angiotensin-aldosterone system (RAAS) also aggravates microinflammation in the kidney [19, 20]. Oxidative stress is another important factor in DKD. Excessive reactive oxygen species (ROS) production in kidney tissues activates inflammation-related signaling pathways such as protein kinase C (PKC), mitogen-activated protein kinase (MAPK), and nuclear factor-*κ*B (NF-*κ*B), which lead to the production of a large number of cytokines and growth factors, hence triggering the deposition of extracellular matrix (ECM) in kidney glomeruli [21]. Proteinuria, a marker of renal lesion, aggravates local microinflammatory responses and interstitial cellular infiltration, leading to overexpression of mesangial matrix protein, thickening of glomerular basement membrane, and glomerulosclerosis [22].

A growing number of reviews present a common consensus that adhesion molecules (e.g., intercellular cell adhesion molecule (ICAM)-1), diverse inflammatory cytokines (e.g., interleukin (IL)-1, IL-6, IL-18, and tumor necrosis factor-alpha (TNF-*α*)), and chemokines (e.g., monocyte chemotactic protein-1 (MCP-1)/chemokine C-C motif ligand 2 (CCL2), C-X3-C motif chemokine (CX3CL1), and CCL5/RANTES) are involved in the pathogenesis of DKD. Activation of the JAK/STAT pathway is also crucial, as it results in activation of NF-kB, a key transcription factor for inflammatory processes in DKD [18, 20, 23, 24]. Recently, there are increasing amounts of research focusing on the NOD-like receptor protein 3 (NLRP3) inflammasome in the onset and development of DKD. Hyperglycemia is also shown to stimulate the expression of NLRP3, resulting in increased secretion of cytokines IL-1*β* and IL-18 [25, 26].

### 2.3. Inflammation May Be the Material Basis of “Heat Evil” in DKD

Diabetes is a state of chronic low-grade inflammation, and Prof. Feng [27] proposed that heat evil might be the key factor for this. Liu et al. [10] pointed out that “heat toxicity” can result in inflammatory reactions, which provided the theoretical basis for treating inflammation with heat-clearing and detoxifying herbs. Wang et al. [11] used the BATMAN-TCM database and found that 11 herbal medicines with heat-clearing property could activate NF-*κ*B, MAPK, and JAK-STAT pathways, through similar modes of action. These studies provide a molecular basis that herbal medicines with heat-clearing function have an anti-inflammatory effect.

Our research, an analysis on the syndrome of 215 DKD patients at Mogensen 3-4 stage, showed that the heat syndrome was one of the main syndromes, and the syndrome score had a positive correlation with glycosylated hemoglobin levels and renal function index. The expressions of serum TNF-a, IL-6, and C-reactive protein (CRP) were also higher in the heat syndrome group. In conclusion, heat evil is pertinent in the progression of DKD and microinflammation may be its molecular basis.

### 2.4. Properties of Herbal Medicines for Heat-Clearing

Ancient physicians summarized herbal properties based on the herbs' therapeutic effects including warm, heat, cool, and cold; the herbal property is an important part of the herbal medicine theory. It was recorded in* Shen Nong's Herbal Classic* that herbs with cool or cold properties can be applied for diseases with heat syndrome presenting with symptoms such as fever, hot flashes, sweating, dry mouth, thirstiness, red tongue, and rapid pulse. In other words, herbs with cool or cold properties possess therapeutic functions such as clearing heat, detoxifying, cooling blood, and nourishing Yin. Chemical components of heat-clearing herbs include organic acids, flavonoids, glycosides, alkaloids, sugars, tannins, amino acids, esters, volatile oil, mineral, and terpenoids, all of which have antibacterial and anti-inflammatory functions, along with antipyretic and immune enhancing functions [28], similar to the concept of clearing heat evil in Chinese medicine.

### 2.5. Application of Herbal Medicines for Heat-Clearing in the Treatment of DKD

The application of TCM with heat-clearing function in DKD has been widely recognized in the field of TCM. Professor Wang [29] proposed that heat evil runs through the whole DKD and can be divided into stagnated heat with the symptoms of bitter taste in mouth, depression, or irritability in the early-microalbuminuria stage; accumulation heat in the mid-macroalbuminuria stage as described above; and the turbid heat with the symptoms of bad odor in mouth, pruritus skin, constipation, and oliguria in the late-ESRD stage. Herbs with heat-clearing function such as* Huangqin*,* Lianqiao, Mudanpi, Xuanshen,* and* Niubangzi *were recommended in the early-microalbuminuria stage. Herbs with heat-clearing function such as* Niubangzi, Haizao, Shuizhi, and Huangkui*, combined with* Huangqin* were applied in the mid-macroalbuminuria stage. Herbs such as* Cansha, *raw* Yiyiren, Huanglian, Banxia, Jiaoshanzhi,* and* Wuzhuyu *were prescribed in the late-ESRD stage.

Professor Wang Bao Kui [30] proposed that heat evil attacking the kidney is the main pathogenic process in DKD. In the early stage, patients present with increased eGFR, slippery pulse, and red tongue, and treatment focuses on clearing heat and cooling blood, using herbs with bitter flavor and cold property to clear heat and drain fire, such as* Huanglian*,* Gegen*,* Shengdihuang*, and* Huangqin*. When macroalbuminuria appears in the disease progression, herbs with the effect of dispelling wind, invigorating spleen, or activating blood circulation, such as* Qingfengteng*,* Chuanshanlong*,* Cangzhu*,* Fried Baizhu*,* Taoren*, and* Danshen*, can be used.

Professor Sheng [31] proposed that heat evil led to heat toxin, a more severe form of heat, as a pathological product in DKD process. Heat toxin invades the renal collaterals and mixes with blood stasis and phlegm, resulting in renal sclerosis. Meng Jianing et al. [7] proposed that heat stasis was important in the pathogenesis of DKD in the middle-late period. Studies about* Flos Abelmoschus Manihot (Malvaceae)* [32], an extract of* Huangkui*, a herb with heat-clearing and damp eliminating properties in the treatment of DKD, had proved that* Flos A. Manihot *significantly decreased proteinuria as well as serum creatinine without gastrointestinal discomfort.

### 2.6. Anti-Inflammation May Be the Material Basis for “Heat-Clearing” in DKD

Literature reports with regard to this aspect will be discussed over three categories, namely: herbal medicine extracts, single herbs, and compound prescriptions.

#### 2.6.1. Extracts of a Certain Herbal Medicine with Heat-Clearing Properties

Paeoniflorin, an extract of* Radix Paeoniae Alba*, was reported to reduce urinary protein excretion rate of db/db mice by inhibiting macrophage infiltration and TLR2/4 signaling pathway [33] in DKD rat models. It could also inhibit the expression of inflammatory factors IL-6, MCP-1, and ICAM-1 by reducing oxidative stress and inhibit phosphorylation of NF-kB p65 both in DKD rat models and mesangial cells stimulated by high glucose [34]. Rheic Acid [35], an extract of* rheum officinale*, decreased the expression of TGF-*β*1 and MCP-1 in mesangial cells cultured in high glucose medium, hence decreasing high glucose-induced inflammatory response and increasing the survival rate of the cells, as compared to the control group.

#### 2.6.2. Single Herb with Heat-Clearing Properties

*Coptis Chinensis*, a herb with bitter flavor and cold property, is commonly prescribed in DM and DKD patients. Guo et al. [36] showed that Coptis Chinensis could decrease the expression of NF-*κ*B, increase the expression of PPAR-*γ*, and alleviate the pathological changes of kidney in STZ-induced diabetic rats.* Huangkui capsules* [37], a patent drug for DKD patients with damp heat syndrome, improved renal function, by decreasing 24 h proteinuria and serum creatinine, and inhibited the expression of hypersensitivity creative protein (Hs-CRP) and TNF-*α*, as shown by an 8-week clinical study involving 90 patients.

#### 2.6.3. Compound Prescriptions with Heat-Clearing Herbal Medicines

*Yangyinqingre* formula [8] decreased serum glucose, insulin resistance, serum creatinine, and proteinuria in DKD models, and this renoprotection function could be related to its action in anti-inflammation, which involves inhibition of expression of TNF-*α*, IL-17, VEGF and CRP. Similar results were reported in* Sanhuangtangshenkang *granule [38] which could reduce serum fasting blood glucose (FBG), HbA1c, MALB, BUN, and CRP and alleviate microinflammatory stated by downregulating the expression of NF-*κ*B, MCP-1, and CCR2 in DKD rats model.

To sum up, herbal medicines with heat-clearing property can inhibit intracellular pathways like NF-*κ*B and JAT/STAT pathways as well as NLRP3 inflammasome, decrease chemokines and adhesion molecules like CCL2 and ICAM-1, and downregulate cytokines such as IL-1*β*, IL-18, and TNF-*α*, hence giving rise to their anti-inflammatory function. That is to say, anti-inflammation may be the material basis of “heat- clearing” in Chinese medicine theory.

### 2.7. Safety

Herbs with heat-clearing property play an important role in ameliorating the progress of DKD by reducing inflammatory reaction, as supported by above studies. Chinese herbal monomers with components similar to chemical drugs are more easily accepted by international researchers and doctors. However, compound formulas are much more commonly applied in TCM prescriptions, but the safety and the mechanism of these prescriptions are questioned due to their unclear components and complexity. Many researchers tried to answer this question [39, 40] and it was found that the safety was associated with individual's body constitution, quality of TCM, overdosage, and self-medication. Moreover, reasonable combination of herbs in compound prescriptions according to TCM theory can alleviate digestive discomfort, which is a common adverse reaction of herbs with heat-clearing property.

## 3. Summary

**Figure 1:**
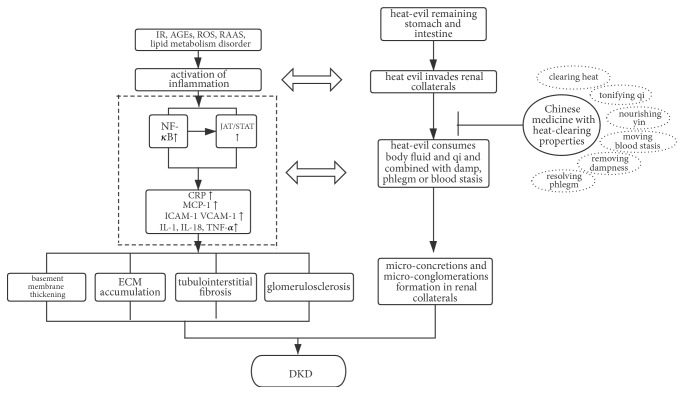
*The corresponding relation between the pathogenesis of TCM theory and Western medicine theory*.* Notes*. IR: insulin resistance; ROS: reactive oxygen species; RAAS: renin-angiotensin-aldosterone system; NF-*κ*B: nuclear factor-*κ*B; JAT/STAST: Janus kinase/signal transducers and activators of transcription; CRP: C-reactive protein; MCP-1: monocyte chemokine-1; ICAM-1: intercellular adhesion molecule 1; VCAM-1: vascular cell adhesion molecule-1;IL: interleukin; TNF-*α*: tumor necrosis factor-*α*; ECM: extracellular matrix.

With the changes in lifestyle and dietary habits, the pathogenesis of DM and DKD has changed in TCM. Heat evil, which can be linked to microinflammation in modern medicine, is essential in the onset and progression of DKD. With the internationalization of TCM culture, the advantages of TCM in the treatment of chronic diseases are increasingly recognized. Herbs with heat-clearing property are beneficial for renal function and ameliorating the progression of DKD by inhibiting inflammatory reactions, as proved by clinical, cellular, and animal studies (Figure 1). Application of heat-clearing medicines provides a new direction for DKD treatment and can be promoted as a supplementary therapy. Our team is conducting a randomized control trial (RCT) study on the heat-clearing medicines in the treatment of DKD, to further provide high-quality medical evidence for the evaluation of the efficacy of heat-clearing herbs on DKD. In addition, herbs with heat-clearing property may have other mechanisms besides anti-inflammation, such as targeting RAAS, reducing oxidative stress, and regulating lipid metabolism disorders, which are important in halting disease progression. Hence, the compatibility and multitarget advantages of TCM prescriptions are worth further exploring and researching.
